# Shape Memory Behavior of Natural *Eucommia ulmoides* Gum and Low-Density Polyethylene Blends with Two Response Temperatures

**DOI:** 10.3390/polym11040580

**Published:** 2019-04-01

**Authors:** Lin Xia, Shuai Chen, Wenxin Fu, Guixue Qiu

**Affiliations:** 1 Key Laboratory of Rubber-Plastics, Ministry of Education/Shandong Provincial Key Laboratory of Rubber-Plastics, School of Polymer Science and Engineering, Qingdao University of Science and Technology, Qingdao 266042, China; xialin@qust.edu.cn (L.X.); cs19911001@163.com (S.C.); 2 Materials Science and Engineering, School of Engineering, University of California at Merced, 5200 North Lake Road, Merced, California 95343, USA; wfu7@ucmerced.edu

**Keywords:** natural *Eucommia ulmoides* gum, DCP dosage, shape memory, cross-linked network, low-density polyethylene

## Abstract

A series of shape memory blends of natural *Eucommia ulmoides* gum (EUG) and low-density polyethylene (LDPE) with a bicontinuous cross-linked structure were prepared by a physical blending method, which could be used in the field of thermal response with two different temperatures. We report the shape memory properties of these blended materials with two response temperatures for the first time. The mechanical, curing, thermal and shape memory properties of the blends were studied in this manuscript. Schematic diagrams are proposed to illustrate the dual shape memory behaviors of the EUG/LDPE blends. Our study focused on observing the relationship between the shape memory behavior and the microscopic crystalline phase states in the blends. In the blends, both the cross-linked network and the LDPE crystalline regions could act as fixed domains, while the crystalline regions of LDPE or EUG could act as the reversible domain. The shape memory properties were mainly determined by the components of the fixed and reversible domains. We focused on the shape memory behavior of blends at 60 °C and 130 °C in this manuscript. The results showed that when the peroxide dicumyl peroxide (DCP) dosage was 1.0 phr, the blends exhibited acceptable shape behavior at 60 °C (R_1f_ = 74.8%, R_1r_ = 63.3%). At the same time, when DCP dosage was 0.4 phr, the shape memory behavior of the blends at 130 °C was good and much better than that at 60 °C (R_2f_ = 91.1%, R_2r_ = 89.4%).

## 1. Introduction

Shape memory polymers (SMPs) are novel smart materials which possess the capacity to maintain temporary shapes and recover their permanent shape under an external stimulus [1], such as temperature [2,3], pH [4,5], light [6,7,8] and an electromagnetic field [9,10]. With the merits of being lightweight, structurally versatile and having large recovery ability [11], SMPs exhibit tremendous advantages over shape memory alloys (SMAs) and shape memory ceramics (SMCs) [12,13]. These advantages provide SMPs with potential applications in aerospace [14], self-healing materials [15,16,17,18], intelligent textiles [19,20] and biomedical devices [21,22,23]. 

Generally, most SMPs are called dual SMPs because of their ability to change into two different shapes. The shape memory mechanism of SMPs can be illustrated as follows. First, the SMP specimen is deformed into a temporary shape above the transition temperature (T_trans_) under mechanical stress. The parameter T_trans_ is vital for SMPs and usually refers to the glass transition temperature (T_g_) for amorphous polymers and the melting temperature (T_m_) for crystalline polymers [24]. Second, when the temperature is cooled to T_trans_, the temporary shape of the specimen is kept under the mechanical stress and fixed after removal of the stress. Finally, the permanent shape of the specimen is recovered by reheating the specimen above T_trans_, which is derived from the entropic elasticity stored in the samples in the stretch process. The shape memory behavior is imparted by two components of the SMPs: the first component is the fixed domain used for sustaining the permanent shape, which might consist of physically or chemically cross-linked points [25]. The second component is the reversible domain used for obtaining the temporary shape, which might consist of crystalline regions or soft segments of SMPs [26]. From a micro perspective, the fixed domain produces an elastic restoring force after material deformation, and the reversible domain freezes or unfreezes the temporary shape after material deformation by crystallization and melting transition. 

Natural *Eucommia ulmoides* gum (EUG) is a biological source of polymers which can be extracted from the *Eucommia Oliv.* plant, an economical and important medicinal plant in China. EUG mainly consists of trans-1, 4-polyisoprene, an isomer of natural rubber, cis-1, 4-polyisoprene. EUG exists in the crystalline state at room temperature due to its ordered trans molecular structure, which makes EUG flexible and plastic [27,28,29]. Partly cross-linked trans-1, 4-polyisoprene has attracted considerable attention for application in SMPs due to its special characteristics, such as remarkable shape recovery stress and large deformability [30,31,32], which are derived from the synergistic effects between crystalline regions and cross-linked networks. In addition, polyethylene (PE) has also exhibited a shape memory effect by cross-linking [33,34,35,36]. A series of shape memory composites have been prepared using thermoplastics and rubber materials as matrix in our research group [37,38]. In this paper, we designed SMP blends with EUG and low-density polyethylene (LDPE) by a simple physical blending method with two response temperatures, which was favored for its merits of simplified operation, high preparation efficiency and material availability. The mechanical, thermal and shape memory properties at different temperatures were investigated. Furthermore, schematic diagrams are proposed to explain the effects of the fixed domains on the shape memory behavior of the blends. These blends could be easily prepared and potentially used in the field of thermal sealing and shape memory annular tubes with two different response temperatures. 

## 2. Experimental Section

### 2.1. Materials

LDPE (type LD100AC) pellets with a melt flow index (MFI) of 2.1 g (10 min)^−1^ were purchased from Sinopec Beijing Yanshan Co., Ltd. (Beijing, China). EUG (99% purity) with a mooney viscosity of 103.3 was extracted from *Eucommia* plants in our lab. Dicumyl peroxide (DCP) with purity of 96% was purchased from Aladdin Co., Ltd. (Shanghai China). Other additives were obtained from commercial sources and used without further purification.

### 2.2. Preparation of EUG/LDPE Blends

The LDPE pellets and EUG were dried in a vacuum oven at 50 °C for 12 h. EUG (40 phr) and LDPE (60 phr) were mixed on a high-temperature open mill at 110 °C for 10 min, followed by adding triallyl isocyanurate (0.5 phr), antioxidant 264 (2 phr) and different dosages of DCP. There was 0.4 phr DCP in formulation A, 0.6 phr DCP in formulation B, 0.8 phr DCP in formulation C and 1.0 phr DCP in formulation D.

### 2.3. Cure Characteristics of Blends

The curing characteristics of the blends were studied with a Monsanto oscillating disc rheometer at 170 °C according to ASTM D-2084-11. The optimal cure time and scorch time were determined from the results in Table 1.

### 2.4. Mechanical Characterization

Vulcanized slabs were prepared by compression molding, and dumbbell-shaped specimens were die-cut according to dimensions in ASTMD 412-type C. The tests were conducted following the ASTM D 412-16 and ASTM D 624-00 procedures. The modulus at 100% and 300%, elongation, tensile strength, tear strength and elongation at break were measured at room temperature using a Lloyd LR10 K tensile testing machine. The gauge length of the dumbbell samples was 25 mm, and the speed of the jaw separation was 500 mm min^−1^.

### 2.5. Differential Scanning Calorimeter (DSC) Measurements

The thermal properties of the blends were determined using a DSC-Q20 (TA Instruments, Newcastle, DE, USA) under a nitrogen atmosphere. The temperature and enthalpy were calibrated with an indium standard. Samples with a mass of 5–10 mg were maintained at 130 °C for 3 min to eliminate their thermal history before they were cooled to −50 °C at 10 °C min^−1^. The samples were subsequently heated to 130 °C at 10 °C min^−1^. The first cooling and subsequent heating traces were recorded for analysis. The degree of crystallinity (X_c_) for each portion of the sample was calculated as follows:

(1) $$X_{c}=\frac{\Delta H_{m}}{\Delta H_{m}{}^{*}}\times 100\%$$

where Δ*H*_m_ and Δ*H*_m_^*^ are the melting enthalpy of a certain polymer portion and its theoretical melting enthalpy (ca. 186.8 J g^−1^ for EUG and ca. 277.1 J g^−1^ for LDPE), respectively.

### 2.6. Shape Memory Effect Analysis

The shape memory properties of the blends were analyzed by a DMA Q800 instrument (TA Instruments, Newcastle, Delaware, USA) in 'Controlled Force' mode. The preload was 0.001 N. Test samples with a thickness of 2.0 mm were cut into rectangular shapes with widths of 4.0 mm and lengths of 30.0 mm. The initial clamp gap was set to be 6.0~8.0 mm. The heating and cooling rates were both 5 °C min^−1^. The procedures for examining the shape memory effect were as follows.

When the cross-linked network and crystalline regions of LDPE acted as the fixed domain, the sample was first maintained isothermally at 60 °C for 5 min to completely melt the crystalline domain of EUG (the initial strain was marked as ɛ_s0_). Second, a mechanical stress of 1.0 MPa was applied, and the sample was cooled to −20 °C at 5 °C min^−1^ to completely freeze the crystalline domain of EUG (ɛ_s1,load_). After removal of the mechanical stress, the sample was maintained isothermally at −20 °C for 5 min (ɛ_s1_). Finally, the sample was reheated to 60 °C at 5 °C min^−1^ and maintained isothermally for 15 min (ɛ_s0,rec_). 

When only the cross-linked network acted as the fixed domain, the sample was first maintained isothermally at 130 °C for 5 min to completely melt the crystalline domain of the EUG/LDPE blend (the initial strain was marked as ɛ_s0_). Second, a mechanical stress of 0.025 MPa was applied, and the sample was cooled to −20 °C at 5 °C min^−1^ to completely freeze the crystalline domain (ɛ_s1,load_). After removal of the mechanical stress, the sample was maintained isothermally for 5 min (ɛ_s1_). Finally, the sample was reheated to 130 °C at 5 °C min^−1^ and maintained isothermally for 15 min (ɛ_s0,rec_). 

The shape fixity ratio (R_1f_ and R_2f_) and shape recovery ratio (R_1r_ and R_2r_) are crucial parameters for SME characterization, both of which can be quantified by the following equations:

(2) $$R_{f}(0\to 1)=\frac{\varepsilon_{1}-\varepsilon_{0}{}_{}}{\varepsilon_{1,load}-\varepsilon_{0}}\times 100\%$$

(3) $$R_{r}(1\to 0)=\frac{\varepsilon_{1}-\varepsilon_{0}{}_{,rec}}{\varepsilon_{1}-\varepsilon_{0}}\times 100\%$$

where ɛ_1,__load_ represents the maximum strain under stress, ɛ_1_ and ɛ_0_ are the strain after cooling and stress removal, and ɛ_0, rec_ is the recovered strain.

## 3. Results and Discussion

In this paper, shape memory EUG/LDPE blends were prepared with different vulcanization degrees using peroxide as a vulcanizing agent, which could vulcanize both the EUG and LDPE phases. The mechanical, thermal and shape memory properties were investigated, which were closely linked to the crystalline regions and the cross-linked network of the EUG/LDPE blends.

### 3.1. Curing Characteristics and Mechanical Properties

The vulcanizing agent Dicumyl peroxide (DCP) was used to cross-link the EUG/LDPE blends. The amount of DCP directly affected the degree of vulcanization of the blends. The effect of DCP content on the curing characteristics of the blends is illustrated in Table 1. T_10_ was the scorch time, which was usually defined as the time for samples’ torque to increase 10 units during the molding process. T_90_ was the time for samples’ torque to increase 90 units during the molding process, which was closely linked with optimum cure time. As evident from Table 1, there were no significant changes in the scorch and curing times with increasing DCP content. M_H_ and M_L_ were maximum torque and minimum torque during the molding process of samples. However, the value of M_H_-M_L_ showed a rising trend with increasing DCP content, which indicated an improvement in the cross-linking density.

Then, the effect of the DCP content on the mechanical properties of the EUG/LDPE blends was investigated, and the results are shown in Table 2. The tensile strength and elongation at break were improved with increasing DCP content, but there was no significant change in the modulus (both 100% and 300%) and the tear strength. The 100% modulus and 300% modulus were the tensile strength when the elongation was 100% and 300%, respectively. The tensile strength was improved from 12.5 MPa to 16.3 MPa. When the DCP content was 0.8 phr and 1.0 phr, the tensile strength of the blends showed a clear increase. The elongation at break increased from 538% to 690%. We analyzed the reasons for this mechanical phenomenon as follows. Based on the vulcanizing mechanism of DCP, the network was generated in both the EUG and LDPE portions. With a fixed blend ratio of EUG/LDPE, the cross-linked network was enhanced with the increase of DCP content, which was accompanied by the destruction of the crystalline regions of the blends. The increase of the cross-linking structure and the destruction of the crystalline structure have a competitive relationship with the properties of the blends. The increase in the tensile strength indicated that the cross-linking structure played a leading role compared to the destruction of crystalline regions of the blends. As the amount of DCP increased, the crystalline structure was seriously damaged, especially the polyethylene portion, which led to an increase in elongation at break.

### 3.2. DSC Analysis

**Figure 1:**
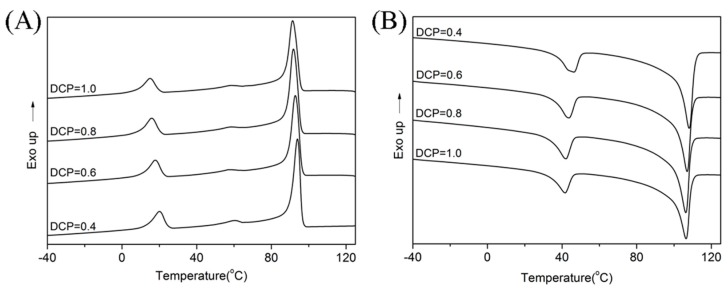
Differential Scanning Calorimeter (DSC) curves of the EUG/LDPE blends with different dicumyl peroxide (DCP) dosage: (**A**) cooling curves and (**B**) heating curves.

The thermal properties of materials are very important for shape memory composites. One of the important parameters, T_trans_, for shape memory materials could be determined from DSC measurements. The melting temperatures and crystallization degrees of the blends could also be obtained from the DSC curves (Figure 1). The peak positions for both the LDPE and EUG portions significantly shifted to the left with the increase in DCP content. The temperature peaks represented the EUG and LDPE portions separately (Table 3). The melting peak area also decreased for the two portions in the blends, which was due to the destruction of their crystalline regions. The degree of crystallinity could be calculated according to Equation (1). We found that the crystallinity of EUG decreased from 11.0% to 7.4%, and the crystallinity of LDPE decreased from 22.8% to 19.0%, as shown in Table 3. Therefore, we deduced that the addition of DCP had a great influence on the microstructure of the blends, especially the cross-linking and crystallization. 

### 3.3. Dual Shape Memory Effect

**Figure 2:**
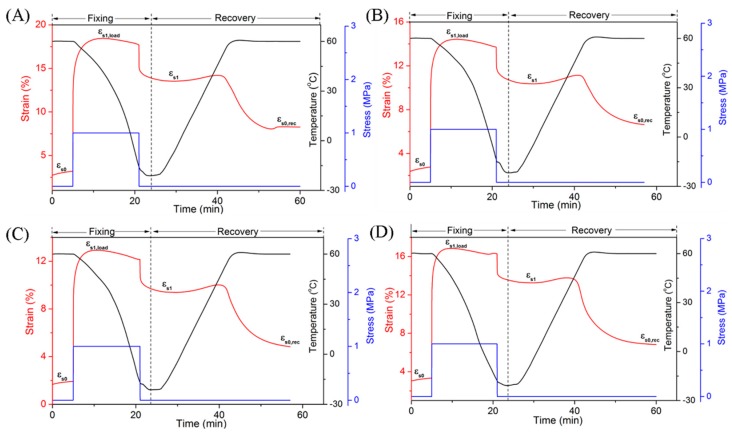
Dual Shape memory properties of the EUG/LDPE blends (T_trans_ = 60 °C) with different DCP content: (**A**) 0.4 phr; (**B**) 0.6 phr; (**C**) 0.8 phr; (**D**) 1.0 phr.

Finally, the shape memory properties of the EUG/LDPE blends at different temperatures were investigated, which might lay a theoretical foundation for the application of the materials. We focused on the shape memory behavior of the blends with maximum recovery temperatures of 60 °C and 130 °C. These two temperatures were chosen according to the melting points of the two basic polymers. The temperature directly determined the states of the polymer phases in the microstructures of the blends, which acted as stationary or reversible phases in the shape memory blends. Two cases of the fixed and reversible domains are discussed as follows. In the first case, when the blends were heated, kept isothermally at 60 °C and cooled to fix the temporary shape, only the crystalline regions of the EUG portion melted. Therefore, in this case, the cross-linked network and crystalline regions of LDPE acted as the fixed domain; meanwhile, the crystalline region of EUG acted as the reversible domain. The shape memory results of the EUG/LDPE blends are shown in Figure 2 and Table 4. The four pictures in Figure 2 include shape fixing and shape recovery processes. The meanings of letters in Figure 2 are explained in the experimental part of shape memory effect analysis. For the dual shape memory effect (SME), the shape fixity ratios (R_1f_) of the blends were maintained at approximately 70%, but there was a slight increase in the shape recovery ratios (R_1r_) from 49.8% to 63.3% with increasing DCP content. We found that the calculation of R_f_ at 60 °C in the DMA test was mainly related to the crystallization of the EUG portion of the blends. The value of R_r_ depended mainly on the entropy elasticity of the stationary phases in the blends during the tensile process. There was a decreasing trend in the red lines in Figure 2 when the mechanical stress was removed, which represented the reduction of stretched length of the samples. This phenomenon was attributed to the disproportion between the fixed and reversible domains in the blends. The temporary shapes were not sufficiently fixed when only the crystalline regions of EUG acted as the reversible domain. The shape memory properties of the blends at 60 °C were not very good, which was mainly determined by the composition and content of crystalline zones, cross-linking structure and other microstructures of the blends. Therefore, the shape memory performance at 60 °C could be improved by adjusting the proportion of EUG and LDPE regions.

**Figure 3:**
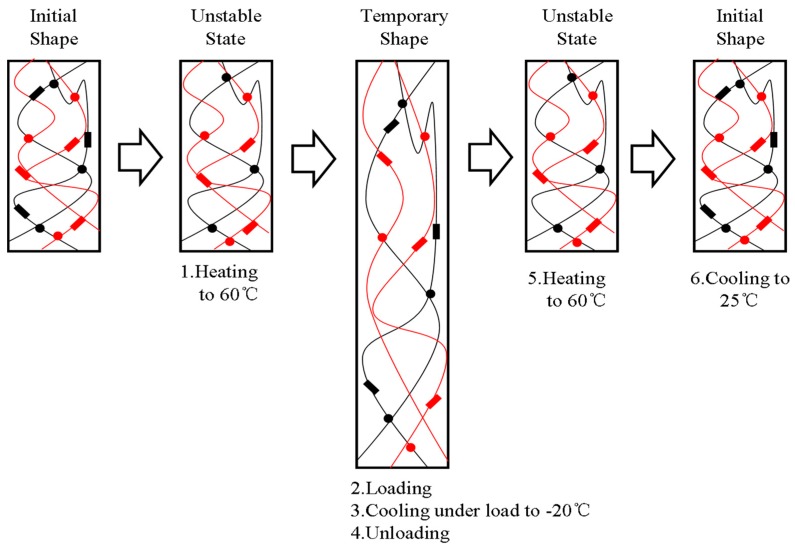
Schematic diagram of the dual SME for the EUG/LDPE blends (T_trans_ = 60 °C).

A schematic diagram was also proposed to illustrate the whole process in Figure 3. In this diagram, the black lines represent the molecular chains of EUG, and the red lines represent the molecular chains of LDPE. Both the red and black rectangles represent the crystalline regions in the blends, and the dots of different colors represent the cross-linking points of the LDPE and EUG portions, respectively. When the blends were heated to 60 °C, only the crystalline regions of EUG melted. A temporary shape could be obtained under mechanical stress and the following cooling process. In addition, the blends could return to their original shapes when they were reheated without the stress. Therefore, the microscopic states of all portions and the shape memory process at deformation temperature 60 °C could be observed from the schematic diagram. 

**Figure 4:**
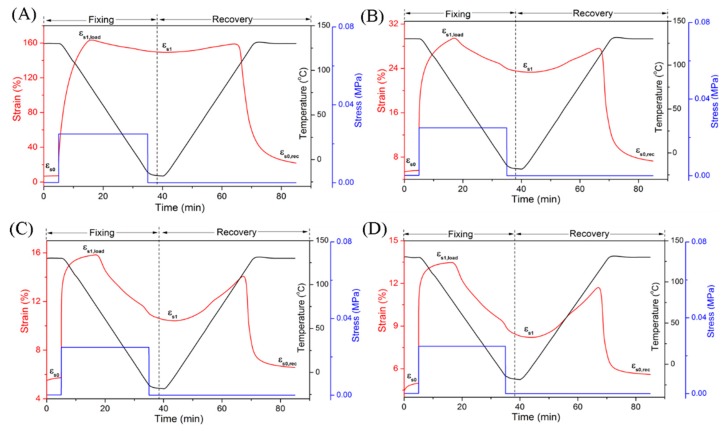
Dual Shape memory properties of the EUG/LDPE blends (T_trans_ = 130 °C) with different DCP content: (**A**) 0.4 phr; (**B**) 0.6 phr; (**C**) 0.8 phr; (**D**) 1.0 phr.

**Figure 5:**
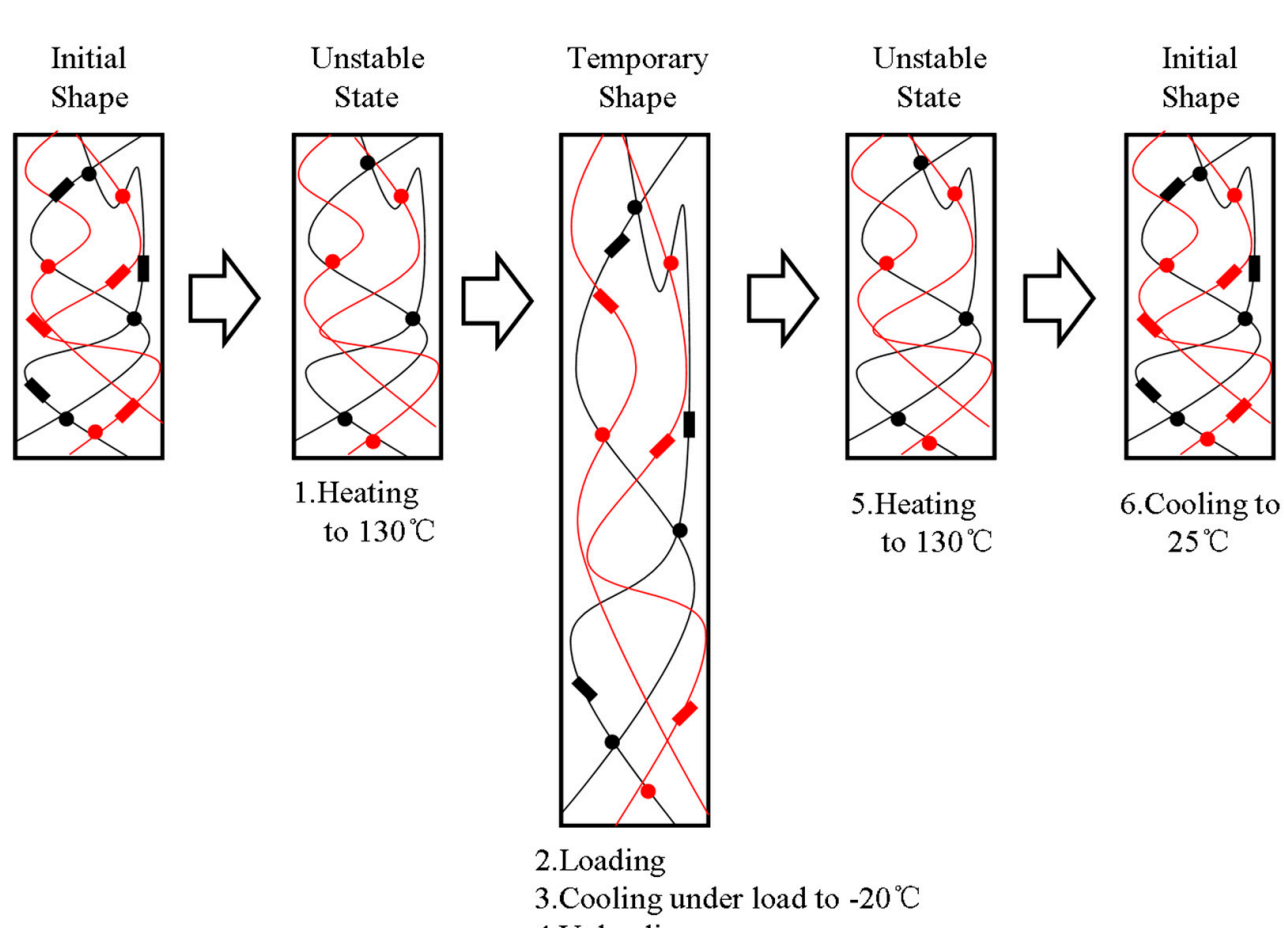
Schematic diagram of the dual SME for the EUG/LDPE blends (T_trans_ = 130 °C).

In the second case, when the samples were heated, kept isothermally at 130 °C and cooled to fix the shape, the crystalline regions of EUG and LDPE both melted, which indicated that the fixed domain consisted of the cross-linked network and the reversible domain consisted of the crystalline regions of two polymers in the blends. The shape memory behavior of the blends at 130 °C was shown in Figure 4, and the detailed data were listed in Table 5. Figure 4 was similar to Figure 2, which also included two parts: shape fixing and shape recovery. The meanings of letters in Figure 5 were also explained in the Experimental part of shape memory effect analysis. An interesting phenomenon was observed. The strain in Figure 4A was much larger than that of Figure 4B–D. In shape memory experiments, the crystalline regions of EUG and LDPE played important roles at 130 °C. The EUG phase with double bonds was cross-linked first when the amount of DCP was low, and the LDPE part had few cross-links. Therefore, the crystalline zone of LDPE was only partially destroyed. This result was also consistent with that of DSC. When the amount of DCP was 0.4 phr, the LDPE part in the blends exhibited great plasticity, resulting in large deformation of blends, close to 160%. The maximum strain decreased gradually from 160% to 30%, 15% and 13% with an increase in DCP content, which reflected the contribution of DCP to the cross-linking of the LDPE part, leading to a reduction in elongation at 130 °C. We found that the EUG/LDPE blends obtained the best dual SME (R_2f_ = 91.1%, R_2r_ = 89.4%) when the DCP dosage was 0.4 phr. However, both the shape fixity ratio and the shape recovery ratio decreased with the increase of DCP content, which changed from 91.1% to 41.7% and 89.4% to 69.2%, respectively.

**Figure 6:**
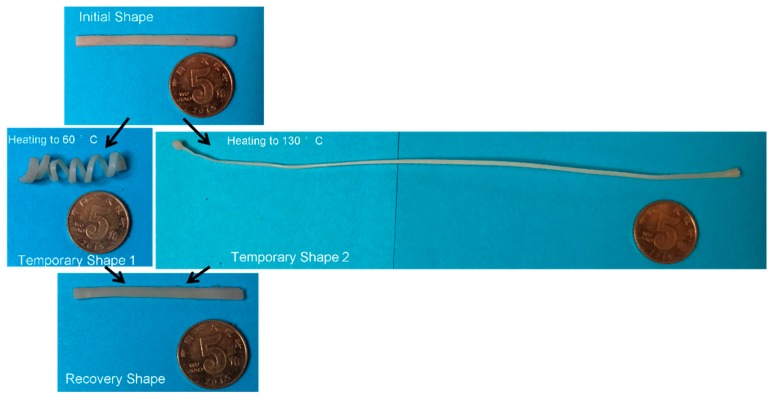
Digital images of the shape memory process for the EUG/LDPE blends.

Similarly, a schematic diagram (Figure 5) was also proposed to illustrate qualitatively the shape memory mechanism of the EUG/LDPE blends in which only the cross-linked network acted as the fixed domain. The black lines represent the molecular chains of EUG, and the red lines represent the molecular chains of LDPE. Both the red and black rectangles represent the crystalline regions in the blends, and the dots with different colors represent the cross-linking points generated in the LDPE and EUG portions. When the samples were heated and kept isothermally at 130 °C, all crystalline regions melted. A temporary shape was formed and fixed when a mechanical stress was applied and the cooling process was preceded. After removing the stress and reheating again, the samples returned to their original shapes. We added digital images in Figure 6. In Figure 6, the sample was first heated to 60 °C, deformed by external stress, and given a temporary shape 1 after the cooling and fixing process. Similarly, the sample was given a temporary shape 2 at 130 °C. When the sample with a temporary shape was reheated again, the material returned to its original shape. 

## 4. Conclusions

We designed and studied the properties of shape memory EUG/LDPE blends with two response temperatures. The mechanical, curing, thermal and shape memory properties of the blends were studied. Schematic diagrams were proposed to illustrate the dual shape memory behaviors of the EUG/LDPE blends. Our study focused on investigating the shape memory behavior of materials at 60 °C and 130 °C under external stress. In the blends, both the cross-linked network and the LDPE crystalline regions could act as fixed domains, while the crystalline regions of LDPE or EUG could act as the reversible domain. The shape memory properties were mainly determined by the components of the fixed and reversible domains. The results showed that when DCP dosage was 1.0 phr, the blends exhibited acceptable shape behavior at 60 °C (R_1f_ = 74.8%, R_1r_ = 63.3%). At the same time, when DCP dosage was 0.4 phr, the shape memory behavior of the blends at 130 °C was good and much better than that at 60 °C (R_2f_ = 91.1%, R_2r_ = 89.4%).

## Figures and Tables

**Table 1:** Curing characteristics of EUG/LDPE blends.

Properties	EUG/LDPE Blends with Different DCP Dosage			
	0.4 phr	0.6 phr	0.8 phr	1.0 phr
M_H_ (dN·m)	2.51	3.60	4.55	4.99
M_L_ (dN·m)	1.02	1.09	1.05	0.98
M_H_- M_L_ (dN·m)	1.49	2.51	3.50	4.01
T_10_ (min)	1.18	1.15	1.20	1.22
T_90_ (min)	11.78	10.40	10.42	10.55
Cure rate index (min^−1^)	9.43	10.81	10.85	10.72

**Table 2:** Mechanical properties of the EUG/LDPE blends.

Properties	EUG/LDPE Blends with Different DCP Dosage			
	0.4 phr	0.6 phr	0.8 phr	1.0 phr
Tensile strength (MPa)	12.5 (±0.3)	13.6 (±0.2)	15.4 (±0.3)	16.3 (±0.3)
100% modulus (MPa)	8.4 (±0.1)	8.3 (±0.1)	8.3 (±0.1)	8.1 (±0.1)
300% modulus (MPa)	9.8 (±0.1)	9.9 (±0.1)	9.8 (±0.1)	9.7 (±0.1)
Elongation at break (%)	538 (±10)	570 (±12)	653(±13)	690 (±12)
Tear strength (kN/m)	68.9(±1.0)	70.6 (±0.8)	70.0 (±1.0)	71.0 (±1.0)

**Table 3:** Crystallinity of the EUG/LDPE blends.

Properties	EUG/LDPE Blends with Different DCP Dosage			
	0.4 phr	0.6 phr	0.8 phr	1.0 phr
T_m (EUG)_ (ºC)	35.40	34.53	33.07	32.75
ΔH_m(EUG)_ (J/g)	20.24	17.95	16.02	13.90
X_c (EUG)_ (%)	11.0	9.6	8.6	7.4
T_m (LDPE)_ (ºC)	101.18	99.84	98.84	98.66
ΔH_m(LDPE)_ (J/g)	63.26	62.83	57.54	52.53
X_c (LDPE)_ (%)	22.8	22.6	20.7	19.0

**Table 4:** Dual shape memory properties of the EUG/LDPE blends (T_trans_ = 60 °C).

Properties	EUG/LDPE Blends with Different DCP Dosage			
	0.4 phr	0.6 phr	0.8 phr	1.0 phr
R_1f_ (%)	70.0	66.3	68.6	74.8
R_1r_ (%)	49.8	46.7	59.0	63.3

**Table 5:** Dual shape memory properties of the EUG/LDPE blends (T_trans_ = 130 °C).

Properties	EUG/LDPE Blends with Different DCP Dosage			
	0.4 phr	0.6 phr	0.8 phr	1.0 phr
R_2f_ (%)	91.1	74.6	47.5	41.7
R_2r_ (%)	89.4	89.2	78.2	69.2
